# Supplementary material for: Fluid-injection-induced earthquakes characterized by hybrid-frequency waveforms manifest the transition from aseismic to seismic slip
Source: Nat Commun. 2021 Nov 25;12:6862. doi: 10.1038/s41467-021-26961-x (PMC8617157; doi:10.1038/s41467-021-26961-x)
Supplement: Supplementary file 1 — Supplementary information [file 41467_2021_26961_MOESM1_ESM.docx]

**Title**

Fluid-injection induced earthquakes characterized by hybrid-frequency waveforms manifest the transition from aseismic to seismic slip

**Authors**

Hongyu Yu^1,2*^, Rebecca M. Harrington^2^, Honn Kao^1,3*^, Yajing Liu^4^, and Bei Wang^1,3^

^1^Geological Survey of Canada, Sidney, British Columbia, Canada, V8L 4B2

^2^Institut für Geologie, Mineralogie und Geophysik, Ruhr-Universität Bochum, Bochum, Germany, 44801.

^3^School of Earth and Ocean Sciences, University of Victoria, Victoria, British Columbia, Canada, V8W 3V6.

^4^ Department of Earth and Planetary Sciences, McGill University, Montréal, Québec, Canada, H3A 0E8.

*Correspondence to: hongyu.yu@rub.de; honn.kao@canada.ca.

**Supplementary Materials include:**

Text S1. Local Signal Check

Figures S1-S7:

Fig. S1. Regional station distribution.

Fig. S2. Alignment of EHWs signals on HHE component on station MG08.

Fig. S3. Representative example of the duration estimation of the low-frequency portion.

Fig. S4. Conceptual 3D plot of EHW ray travel path through velocity structure heterogeneities.

Fig. S5. Consistency check of coda duration and amplitude for representative EHWs in Fig. S4.

Fig. S6. The distance-duration scaling on single station MG08.

Fig. S7. Comparison of spatial distribution between EHWs and typical induced events.

Tables S1-S4:

Table S1. List of EHWs.

Table S2. Hydraulic fracturing injection parameters.

Table S3. Table of active periods of injection for wells W1-W5.

Table S4. Table of modeling parameters.

**Text S1 Local Signal check**

We check several aspects to confirm that EHWs indeed occur in close proximity to the HF well. Firstly, the broadband portion of the EHW waveforms has both visible P- and S-wave arrivals that suggest small (< 5 km) source-station distances (e.g., Fig. 1e). Moreover, the short duration of the low-frequency portion of the waveforms lasts approximately ~10 s, also consistent with a local seismic signal. In contrast, if the EHWs had a teleseismic origin with attenuated (and possibly indistinguishable phase arrivals due to low signal-to-noise ratio, SNR), the apparent waveform duration would be expected to exceed 100 s, as a teleseismic event with an epicentral distance of 500 km or greater would have an S-P time longer than 100 s. Regional events with S-P times of tens of seconds could also be mistaken for EHW signals. We therefore attempt to exclude this possibility by cross-checking the regional earthquake catalogue reported by NRCan (Natural Resources Canada). We found no temporally coincident events listed in the NRCan catalog at distances within 500 km. Finally, we check to confirm that the EHW P-/S-wave arrivals are consistent with the theoretically predicted arrival times at regional stations NBC5 (source-receiver distance ~35 km), NBC6 (~170 km) and NBC4 (~215 km) based on our presumed hypocentral locations close to HF1 (Fig. S1). All of the above checks suggest that EHWs are from local sources near the horizontal wells of HF1. Furthermore, waveforms exhibit similar characteristics only at MG stations with the low-frequency portion of the waveform indicating retrograde particle motion. Dispersion effects and low SNR (<2) at more distant stations (NBC5, NBC6 and NBC4) make it difficult to identify the low-frequency portion of the waveform.


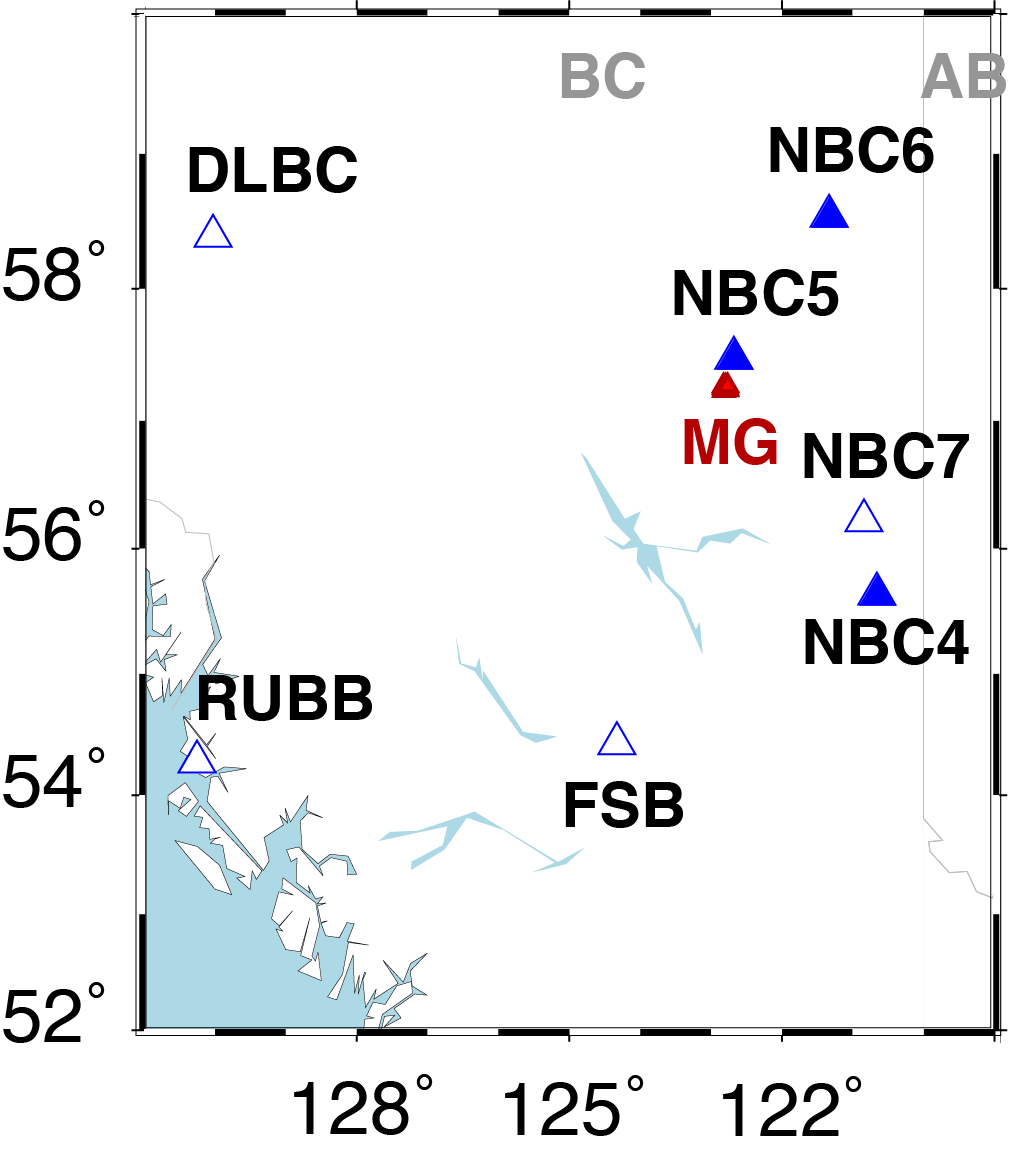


**Fig. S1**. **Regional station distribution.** Red triangle: MG array. Blue solid/empty triangles: stations with/without clear phase arrivals of EHWs (see Text S1).


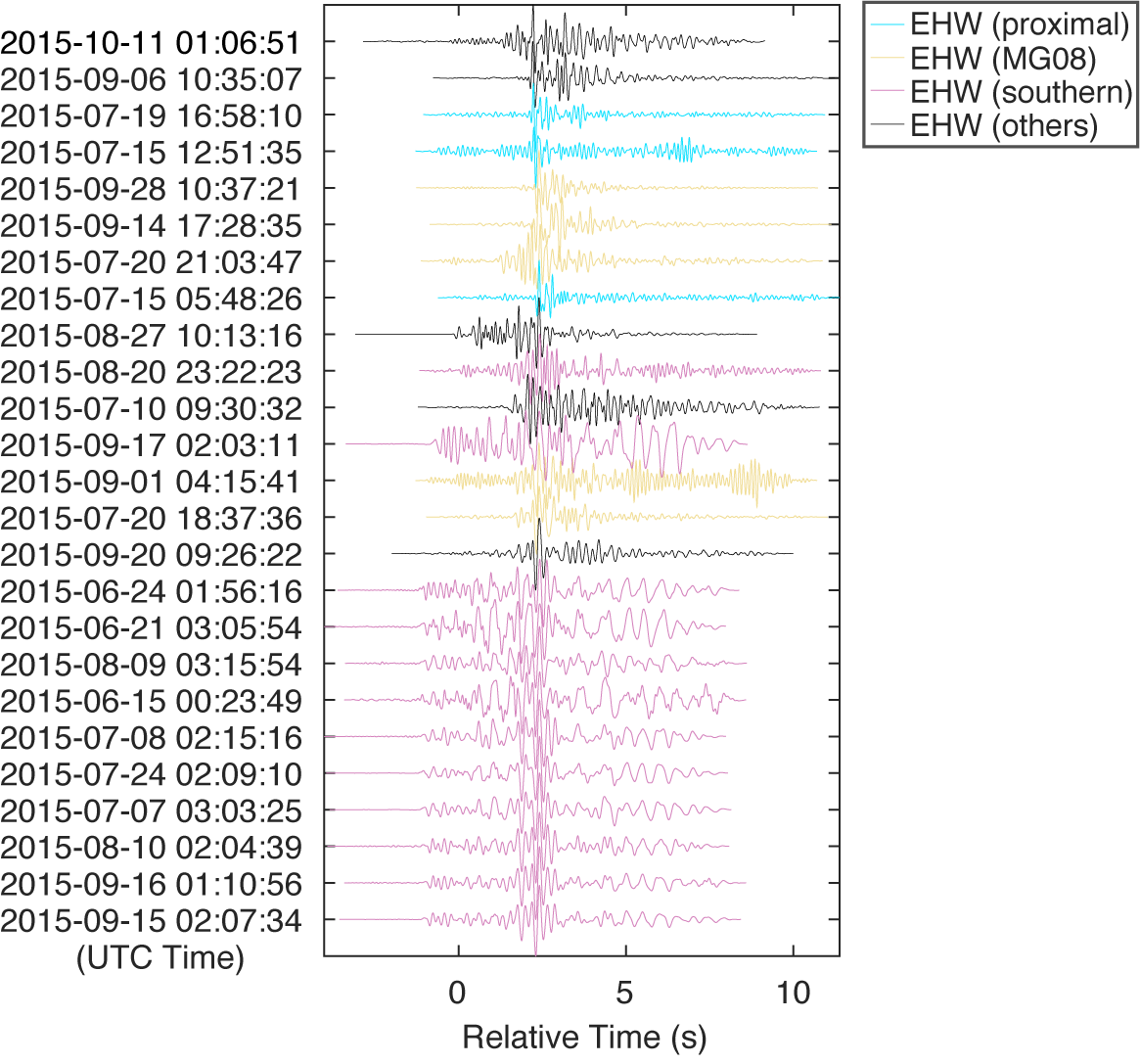


**Fig. S2. Alignment of EHWs signals on HHE component recorded by station MG08.**
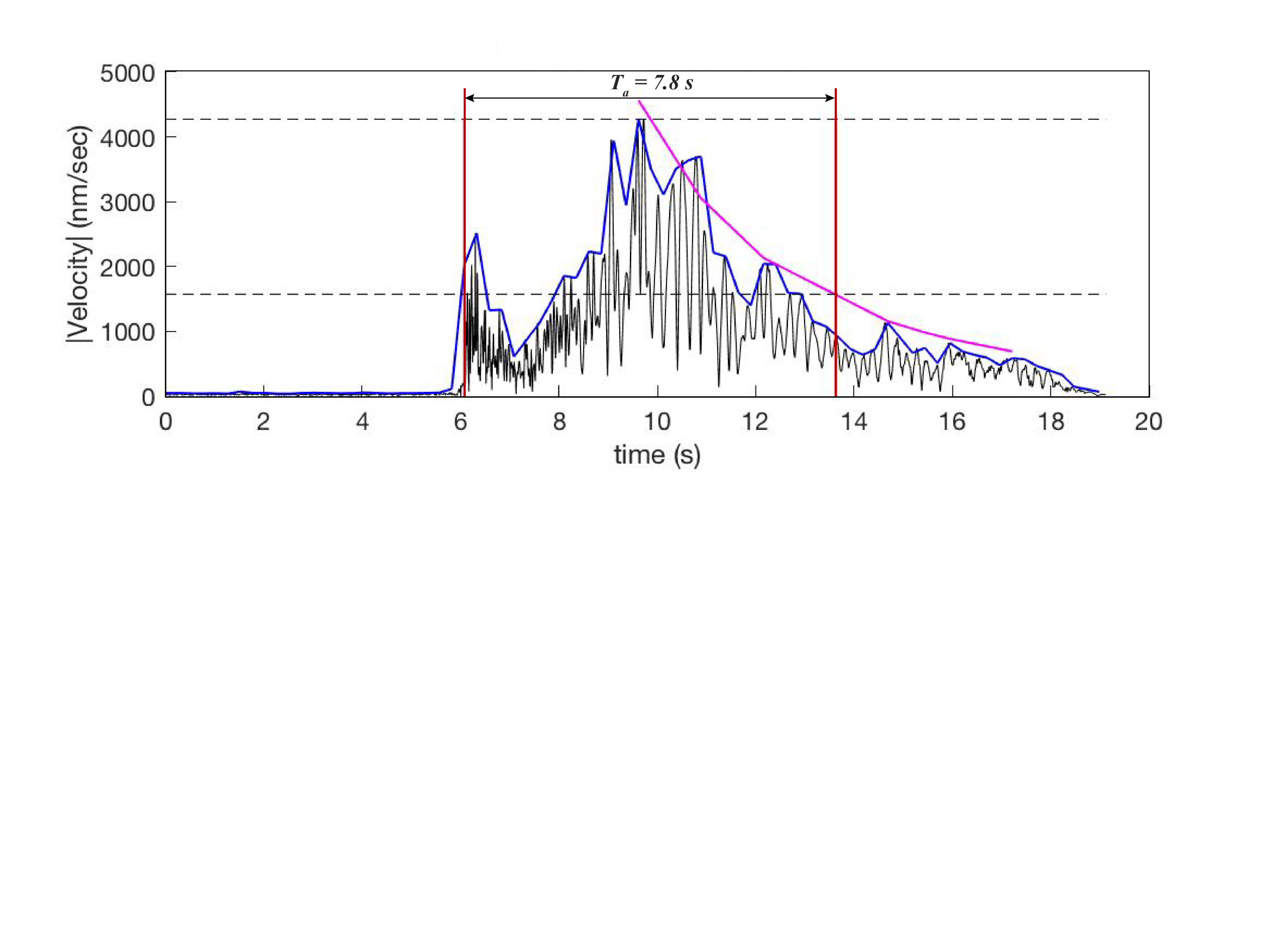


**Fig. S3. Representative example of the duration estimation of the low-frequency coda of an EHW event.** The time between the P-arrival and the time at which the amplitude envelope decays to *e^-1^* of the peak value (bracketed by the vertical red lines) provides an estimate of the duration, *T_a_*, as described in the main text. *T_a_* primarily represents the duration of the low-frequency portion of the waveform. The example shown here has a coda duration of 7.8 seconds.


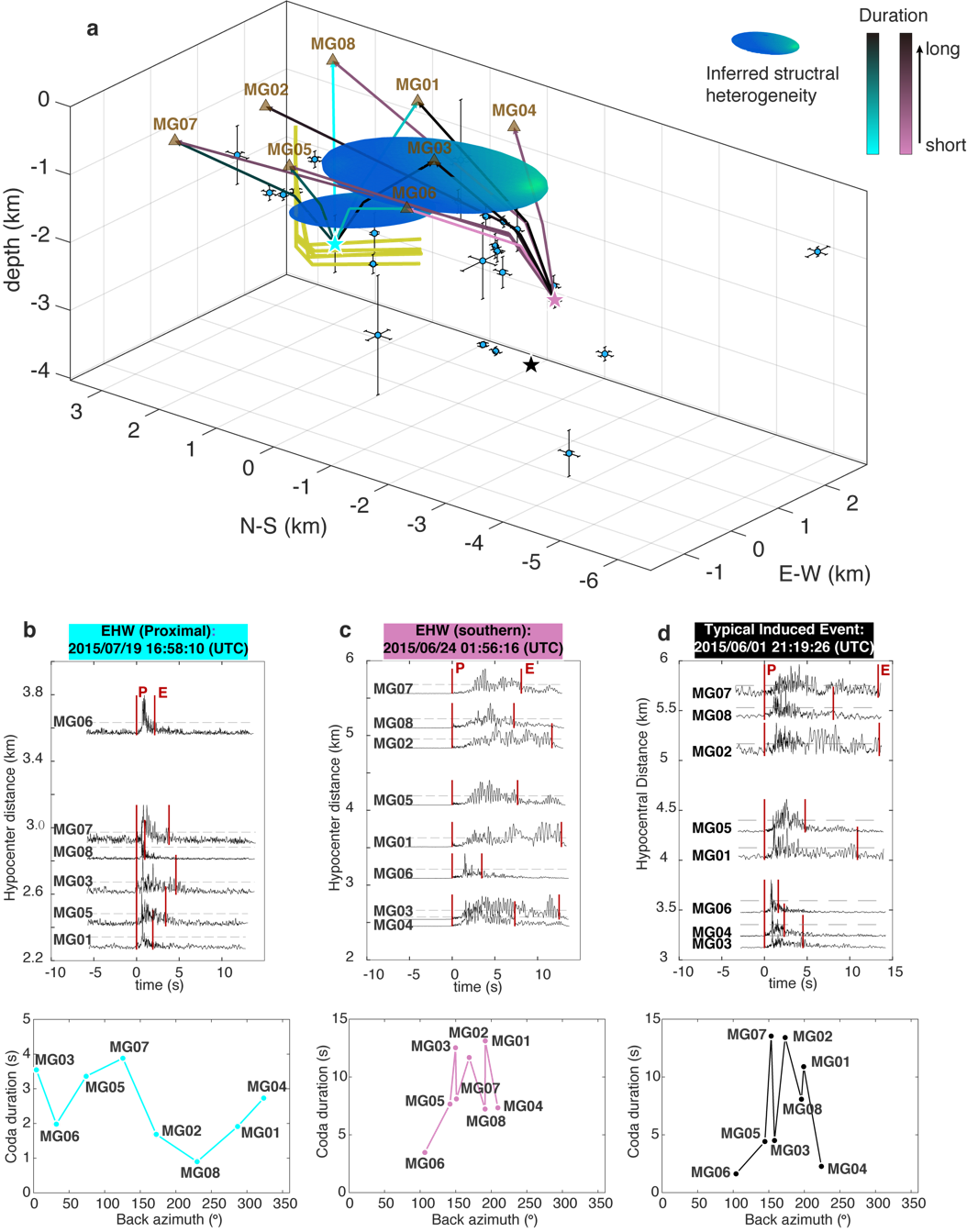


**Fig. S4.** (**a**) Conceptual 3D plot of EHW ray travel path through velocity structure heterogeneities. Blue dots: EHWs. Turquoise/purple stars: example EHWs for a cluster near the wellbore and a cluster south of the wellbore. Black star: source location of typical induced event marked in Fig. 2b. Solid curve: ray path geometry calculated using seismic velocity extracted from CRUST 1.0 (*65*). The color of the ray paths refers to the coda duration, with color bars on the bottom right. Blue ellipsoids: inferred volumes of velocity heterogeneities. Thick yellow curve: hydraulic fracturing well geometry. (**b-d**) Top: envelope record sections layout with hypocenter distances for the three representative events marked as stars in (a). The onset and termination of the signals are marked following the same procedure detailed in Fig. S3. Bottom: diagram of coda duration variation versus station-to-epicenter back azimuth.


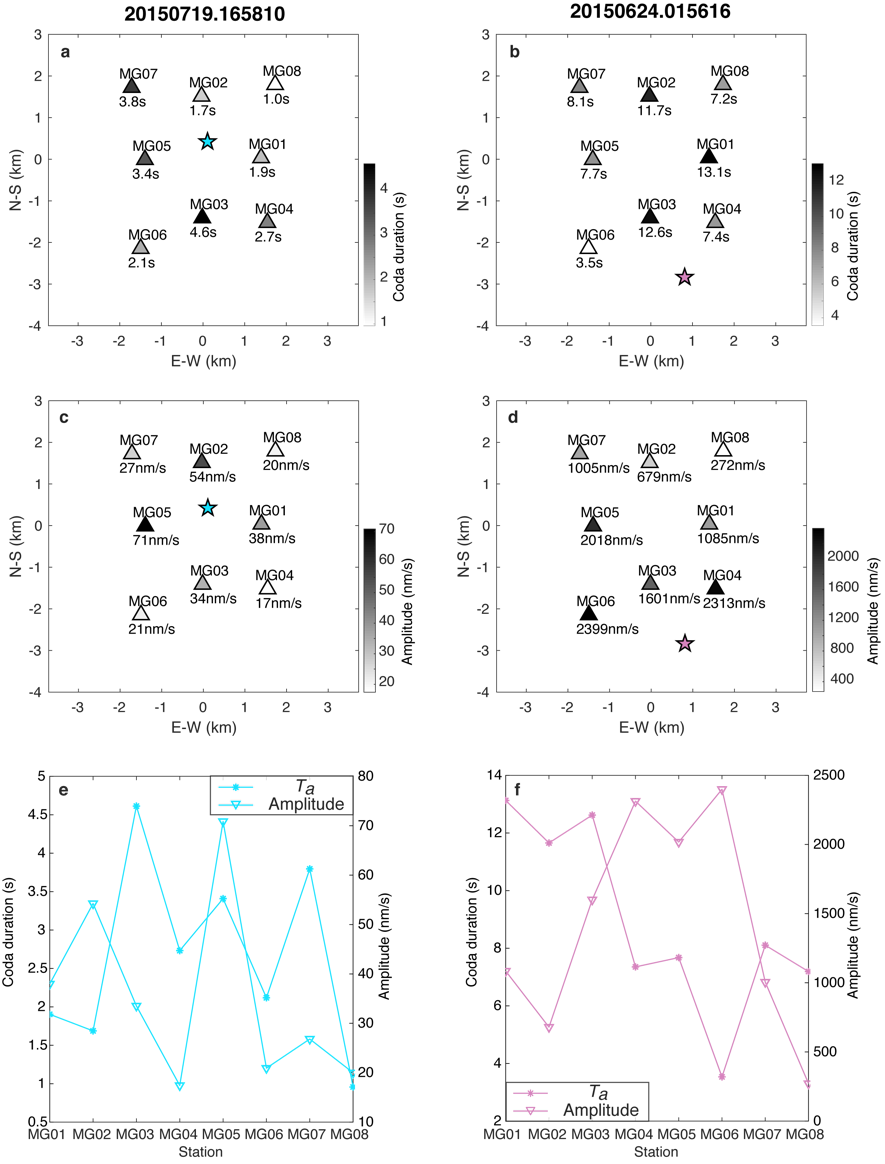


**Fig. S5. Consistency check of coda duration and amplitude of representative EHWs in Fig. S4.** (**a**) Coda duration ($T_{a}$) of the well-proximal EHW example. Triangles: MG stations. Station name and $T_{a}$ values are marked above and below the triangle, respectively. The color of the station also indicates the value of $T_{a}$, with grayscale on the bottom right. (**b**) same as (a), but for an EHW from the southern cluster. (**c-d**) show the amplitude of low-frequency coda using the grayscale on the right of each panel. The comparison between the $T_{a}$ and amplitude are summarized for (**e**) the well-proximal EHW and (**f**) the southern-cluster EHW, respectively. The anticorrelation between $T_{a}$ and amplitude imply a scattering, rather than a radiation pattern effect.

**Fig. S6. The distance-duration scaling on single station MG08.** Each dot represents one EHW. A clear positive correlation exists between the hypocenter distance and coda duration ($T_{a}$).


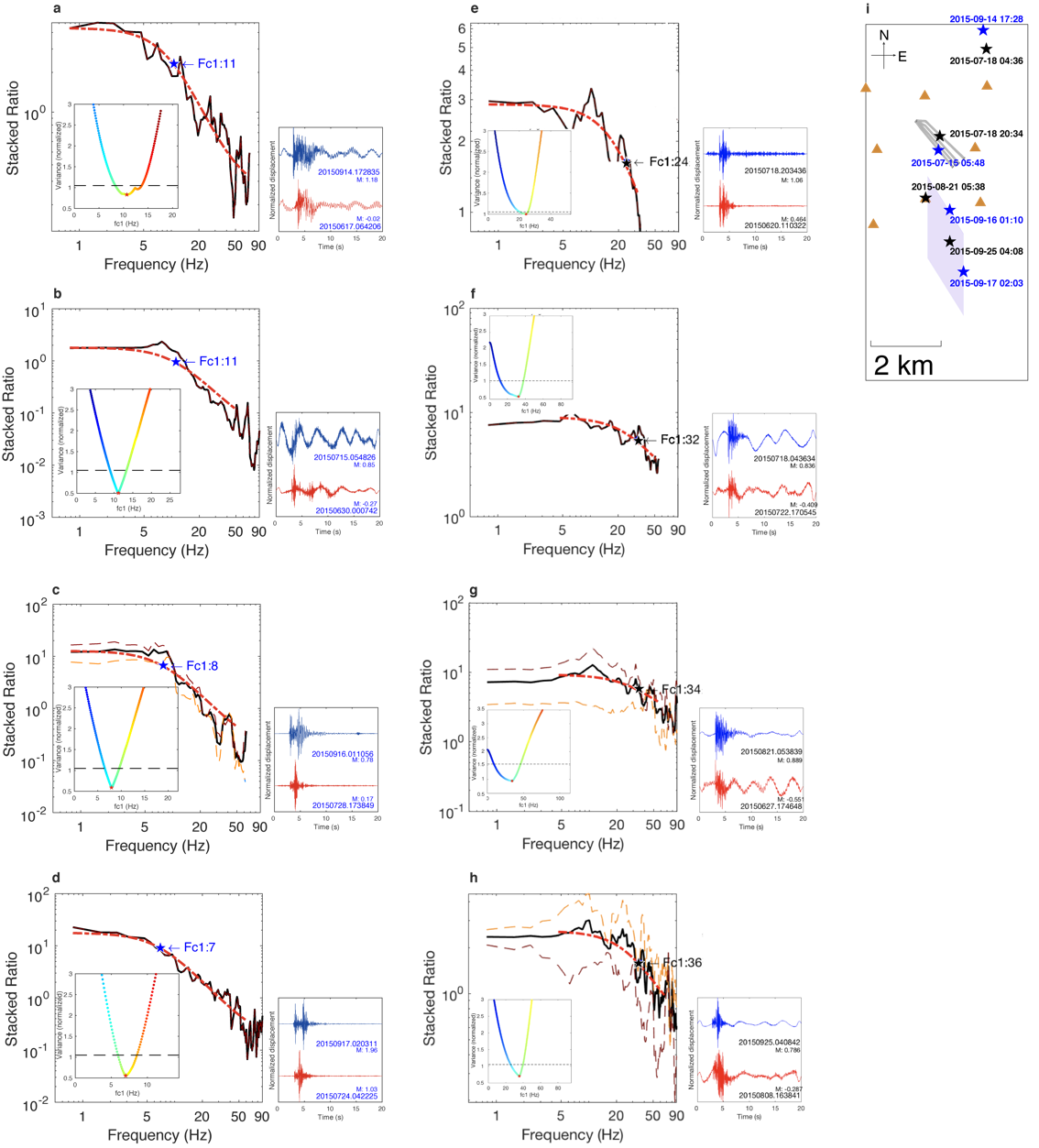


**Fig. S7. Representative examples of spectral ratio fitting for EHWs (a-d) and for typical induced events (e-h).** We provide four representative examples of each type of signal located near station MG08: (**a, e**), near the wellbore: (**b, f**), at shallow depth within the southern structure: (**c, g**), and at greater depth within the southern structure: (**d, h**), respectively. Black line: stacked event pair spectral ratio. Red line: Brune model fit with optimal *f_c_*. Short dashed lines: spectral ratio curves obtained from more than one station. Bottom right panel: Normalized displacement of two events. The event ID in the waveform panels corresponds to the catalog. Bottom left inset: Variance of spectral ratio fit determined by perturbing the corner frequency value of the main event *f_c1_*. Dashed line: 5% increase of fit variance. Star: optimal *f_c1_*. Event locations are specifically marked in (**i**). The EHWs clearly exhibit lower corner frequency values compared to the typical induced events at roughly the same hypocentral location, confirming that the dataset is capable of resolving high corner frequency values and that the low EHW corner frequency values are not the result of observational constraints.

**Table S1.** List of EHWs. Longitude and latitude are in degrees E and N. The units for focal depth, error of longitude (Elon), error of latitude (Elat) and error of depth (Ez) are all km.

| yyyymmdd | hour:min:sec | longitude | latitude | depth | Elon | Elat | Ez |
| --- | --- | --- | --- | --- | --- | --- | --- |
| 20150615 | 00:23:49.050 |  |  |  |  |  |  |
| 20150616 | 01:37:56.800 |  |  |  |  |  |  |
| 20150621 | 03:05:54.490 | -122.7785 | 57.2257 | 1.85 | 0.08 | 0.10 | 0.15 |
| 20150624 | 01:56:16.180 | -122.7820 | 57.2240 | 1.93 | 0.09 | 0.10 | 0.12 |
| 20150628 | 00:52:48.740 |  |  |  |  |  |  |
| 20150707 | 03:03:25.470 | -122.7842 | 57.2289 | 1.00 | 0.04 | 0.10 | 0.32 |
| 20150708 | 02:15:16.400 | -122.7852 | 57.2306 | 0.92 | 0.04 | 0.08 | 0.08 |
| 20150710 | 09:30:31.560 | -122.8187 | 57.2355 | 2.06 | 0.23 | 0.22 | 0.88 |
| 20150715 | 05:48:26.300 | -122.7897 | 57.2491 | 2.02 | 0.06 | 0.06 | 0.14 |
| 20150715 | 12:51:34.930 | -122.7856 | 57.2507 | 1.70 | 0.07 | 0.06 | 0.30 |
| 20150719 | 16:58:10.440 | -122.7937 | 57.2533 | 1.77 | 0.07 | 0.08 | 0.42 |
| 20150720 | 18:37:36.360 | -122.7630 | 57.2730 | 2.22 | 0.09 | 0.09 | 0.06 |
| 20150720 | 21:03:47.080 | -122.7697 | 57.2742 | 2.15 | 0.10 | 0.12 | 0.11 |
| 20150724 | 02:09:09.720 | -122.7948 | 57.2161 | 3.69 | 0.08 | 0.17 | 0.34 |
| 20150725 | 02:37:12.830 | -122.7858 | 57.2305 | 1.65 | 0.08 | 0.14 | 0.23 |
| 20150726 | 02:18:54.900 |  |  |  |  |  |  |
| 20150809 | 03:15:53.580 |  |  |  |  |  |  |
| 20150810 | 02:04:38.750 | -122.7868 | 57.2309 | 1.33 | 0.05 | 0.09 | 0.16 |
| 20150815 | 14:15:37.080 | -122.7930 | 57.2284 | 2.60 | 0.04 | 0.09 | 0.06 |
| 20150820 | 23:22:22.740 | -122.7863 | 57.2334 | 1.56 | 0.44 | 0.23 | 0.56 |
| 20150827 | 10:13:15.550 | -122.7828 | 57.2388 | 0.92 | 0.05 | 0.08 | 0.17 |
| 20150901 | 04:15:41.050 | -122.7671 | 57.2732 | 2.20 | 0.07 | 0.07 | 0.08 |
| 20150906 | 10:35:07.150 | -122.7507 | 57.2526 | 1.39 | 0.20 | 0.11 | 0.89 |
| 20150914 | 17:28:35.280 | -122.7685 | 57.2798 | 1.79 | 0.12 | 0.18 | 0.43 |
| 20150915 | 02:07:34.120 | -122.7857 | 57.2317 | 1.30 | 0.04 | 0.08 | 0.18 |
| 20150916 | 01:10:56.060 | -122.7843 | 57.2338 | 0.96 | 0.04 | 0.08 | 0.17 |
| 20150917 | 02:03:11.060 | -122.7778 | 57.2180 | 2.63 | 0.11 | 0.13 | 0.10 |
| 20150920 | 09:26:22.430 | -122.7618 | 57.1917 | 0.64 | 0.23 | 0.15 | 0.07 |
| 20150928 | 10:37:21.010 | -122.7582 | 57.2722 | 1.83 | 0.10 | 0.10 | 0.12 |
| 20151011 | 01:06:50.880 |  |  |  |  |  |  |
| 20151013 | 01:53:24.530 | -122.7914 | 57.2311 | 2.62 | 0.08 | 0.08 | 0.04 |

**Table S2. Hydraulic fracturing injection parameters.** The end-point locations of each well interval are indicated as (lon1, lat1, dep1) and (lon2, lat2, dep2). The operation hours are calculated from initial hour 2015/07/10 00:00:00.

| well # | stage # | lon1 | lat1 | dep1 (km) | lon2 | lat2 | dep2 (km) | injection rate (m3/s) | initial hour | terminate hour |
| --- | --- | --- | --- | --- | --- | --- | --- | --- | --- | --- |
| 30515 | 1 | -122.7843 | 57.2458 | -2.03 | -122.7831 | 57.2450 | -2.02 | 8.93 | 50.75 | 53.25 |
| 30515 | 2 | -122.7863 | 57.2471 | -2.03 | -122.7850 | 57.2462 | -2.03 | 6.88 | 69 | 72 |
| 30515 | 3 | -122.7883 | 57.2484 | -2.03 | -122.7869 | 57.2475 | -2.03 | 6.39 | 91.25 | 94.25 |
| 30515 | 4 | -122.7902 | 57.2497 | -2.03 | -122.7889 | 57.2488 | -2.03 | 6.57 | 109 | 113 |
| 30515 | 5 | -122.7922 | 57.2510 | -2.03 | -122.7909 | 57.2501 | -2.03 | 6.25 | 128.25 | 132.75 |
| 30515 | 6 | -122.7962 | 57.2536 | -2.03 | -122.7949 | 57.2527 | -2.03 | 7.37 | 211 | 213.5 |
| 30515 | 7 | -122.7982 | 57.2549 | -2.03 | -122.7968 | 57.2540 | -2.03 | 7.91 | 240 | 242.25 |
| 30516 | 1 | -122.7830 | 57.2462 | -1.85 | -122.7819 | 57.2456 | -1.85 | 8.69 | 48 | 50.5 |
| 30516 | 2 | -122.7850 | 57.2475 | -1.86 | -122.7836 | 57.2467 | -1.85 | 7.10 | 63.5 | 66.5 |
| 30516 | 3 | -122.7870 | 57.2488 | -1.86 | -122.7856 | 57.2479 | -1.86 | 6.38 | 80 | 83 |
| 30516 | 4 | -122.7890 | 57.2500 | -1.86 | -122.7877 | 57.2492 | -1.86 | 7.08 | 105.75 | 108.5 |
| 30516 | 5 | -122.7910 | 57.2513 | -1.86 | -122.7897 | 57.2504 | -1.86 | 6.37 | 124.5 | 127.5 |
| 30516 | 6 | -122.7931 | 57.2525 | -1.86 | -122.7917 | 57.2517 | -1.86 | 8.10 | 154 | 158 |
| 30516 | 7 | -122.7951 | 57.2538 | -1.86 | -122.7937 | 57.2529 | -1.86 | 8.64 | 172.25 | 174.5 |
| 30516 | 8 | -122.7971 | 57.2551 | -1.86 | -122.7958 | 57.2542 | -1.86 | 7.43 | 225 | 228 |
| 30517 | 1 | -122.7815 | 57.2467 | -1.97 | -122.7802 | 57.2459 | -1.97 | 9.38 | 32.25 | 34.75 |
| 30517 | 2 | -122.7836 | 57.2480 | -1.97 | -122.7821 | 57.2471 | -1.97 | 9.19 | 84 | 87 |
| 30517 | 3 | -122.7856 | 57.2492 | -1.97 | -122.7842 | 57.2484 | -1.97 | 10.72 | 96 | 97.75 |
| 30517 | 4 | -122.7877 | 57.2505 | -1.97 | -122.7863 | 57.2496 | -1.97 | 9.50 | 115.25 | 117.25 |
| 30517 | 5 | -122.7897 | 57.2517 | -1.97 | -122.7883 | 57.2509 | -1.97 | 5.64 | 144 | 147.75 |
| 30517 | 6 | -122.7918 | 57.2530 | -1.97 | -122.7904 | 57.2521 | -1.97 | 7.18 | 163.25 | 165.75 |
| 30517 | 7 | -122.7939 | 57.2542 | -1.97 | -122.7925 | 57.2534 | -1.97 | 13.94 | 194 | 196 |
| 30517 | 8 | -122.7959 | 57.2555 | -1.97 | -122.7946 | 57.2546 | -1.97 | 5.83 | 229.5 | 233.5 |
| 30518 | 1 | -122.7787 | 57.2476 | -2.02 | -122.7778 | 57.2471 | -2.02 | 7.57 | 38.5 | 42 |
| 30518 | 2 | -122.7801 | 57.2483 | -2.02 | -122.7791 | 57.2478 | -2.02 | 7.60 | 55.5 | 58.5 |
| 30518 | 3 | -122.7815 | 57.2491 | -2.02 | -122.7805 | 57.2486 | -2.02 | 8.19 | 72.5 | 75 |
| 30518 | 4 | -122.7829 | 57.2498 | -2.02 | -122.7820 | 57.2493 | -2.02 | 10.14 | 99.25 | 101.5 |
| 30518 | 5 | -122.7843 | 57.2506 | -2.02 | -122.7833 | 57.2501 | -2.02 | 7.78 | 117.5 | 120 |
| 30518 | 6 | -122.7858 | 57.2513 | -2.02 | -122.7848 | 57.2508 | -2.02 | 7.41 | 136.5 | 139.5 |
| 30518 | 7 | -122.7872 | 57.2521 | -2.02 | -122.7862 | 57.2516 | -2.02 | 7.45 | 158 | 161.5 |
| 30518 | 8 | -122.7886 | 57.2528 | -2.02 | -122.7876 | 57.2523 | -2.02 | 8.07 | 176.5 | 179 |
| 30518 | 9 | -122.7900 | 57.2536 | -2.03 | -122.7890 | 57.2531 | -2.03 | 6.10 | 187 | 190 |
| 30518 | 10 | -122.7914 | 57.2543 | -2.03 | -122.7905 | 57.2538 | -2.03 | 7.00 | 203 | 206 |
| 30518 | 11 | -122.7928 | 57.2551 | -2.03 | -122.7919 | 57.2546 | -2.03 | 4.82 | 215.5 | 219.5 |
| 30518 | 12 | -122.7942 | 57.2558 | -2.03 | -122.7933 | 57.2553 | -2.03 | 7.73 | 235 | 237.75 |
| 30518 | 13 | -122.7957 | 57.2566 | -2.03 | -122.7947 | 57.2561 | -2.03 | 5.69 | 242.5 | 245.5 |
| 30519 | 1 | -122.7778 | 57.2473 | -1.84 | -122.7776 | 57.2472 | -1.84 | 8.14 | 36.25 | 38.5 |
| 30519 | 2 | -122.7793 | 57.2481 | -1.84 | -122.7783 | 57.2475 | -1.84 | 8.23 | 59.5 | 62.25 |
| 30519 | 3 | -122.7808 | 57.2488 | -1.84 | -122.7798 | 57.2483 | -1.84 | 10.65 | 76.25 | 78.5 |
| 30519 | 4 | -122.7823 | 57.2496 | -1.84 | -122.7813 | 57.2491 | -1.84 | 6.71 | 103.5 | 106.5 |
| 30519 | 5 | -122.7839 | 57.2504 | -1.84 | -122.7828 | 57.2499 | -1.84 | 8.40 | 120.25 | 122.5 |
| 30519 | 6 | -122.7854 | 57.2512 | -1.84 | -122.7843 | 57.2507 | -1.84 | 8.48 | 140.25 | 142.5 |
| 30519 | 7 | -122.7869 | 57.2520 | -1.84 | -122.7858 | 57.2514 | -1.84 | 6.19 | 154 | 157 |
| 30519 | 8 | -122.7884 | 57.2527 | -1.84 | -122.7873 | 57.2522 | -1.84 | 8.08 | 165.75 | 168 |
| 30519 | 9 | -122.7914 | 57.2543 | -1.84 | -122.7904 | 57.2538 | -1.84 | 7.62 | 182 | 184.5 |
| 30519 | 10 | -122.7899 | 57.2535 | -1.84 | -122.7889 | 57.2530 | -1.84 | 11.30 | 190 | 192 |
| 30519 | 11 | -122.7929 | 57.2551 | -1.84 | -122.7919 | 57.2546 | -1.84 | 8.19 | 207 | 209.5 |
| 30519 | 12 | -122.7944 | 57.2559 | -1.84 | -122.7934 | 57.2553 | -1.84 | 7.26 | 219 | 222 |
| 30519 | 13 | -122.7959 | 57.2567 | -1.85 | -122.7949 | 57.2561 | -1.84 | 6.72 | 247 | 250 |

**Table S3. Table of active periods of injection for wells W1-W5.**

| well pad # | well # | longitude | latitude | fracturing (year/month) |
| --- | --- | --- | --- | --- |
| W1 | 28452 | 122.801406 | 57.256108 | 2013/09 |
|  | 28514 | 122.801272 | 57.256161 | 2013/09 |
|  | 28885 | 122.801139 | 57.256214 | 2013/09 |
| W2 | 28719 | 122.812083 | 57.294278 | 2014/01 |
|  | 28972 | 122.811953 | 57.294333 | 2014/01 |
|  | 29348 | 122.811822 | 57.294389 | 2014/01 |
| W3 | 26739 | 122.737564 | 57.285769 | 2014/01 |
|  | 27588 | 122.737728 | 57.285756 | 2014/02 |
|  | 28976 | 122.737892 | 57.285742 | 2014/02 |
| W4 | 29656 | 122.769961 | 57.210292 | 2014/07 |
|  | 29718 | 122.769808 | 57.210328 | 2014/07 |
|  | 29785 | 122.769656 | 57.210361 | 2014/07 |
| W5 | 30515 | 122.800475 | 57.256483 | 2015/07 |
|  | 30516 | 122.800342 | 57.256536 | 2015/07 |
|  | 30517 | 122.800208 | 57.256589 | 2015/07 |
|  | 30518 | 122.800075 | 57.256644 | 2015/07 |
|  | 30519 | 122.799942 | 57.256697 | 2015/07 |

**Table S4. Table of modeling parameters.** Velocity model is from CRUST 1.0 with a 1° by 1° grid centered at (57.5°N, 122.5°W) *(65)*. The values of rock and hydrogeological properties are set following *ref. 78*.

|  | Layer 1 | Layer 2 | Layer 3  (shale) | Layer 4 | Layer 5  (basement) |
| --- | --- | --- | --- | --- | --- |
| Depth (km) | 0.0~1.0 | 1.0~1.8 | 1.8~2.1 | 2.1~2.8 | 2.8~5 |
| Biot-Willis | 0.7 | 0.7 | 0.7 | 0.7 | 0.7 |
| P-wave velocity (m/s) | 2500 | 4600 | 4600 | 4600 | 6300 |
| S-wave velocity (m/s) | 1070 | 2590 | 2590 | 2590 | 3630 |
| Bulk Density $\rho_{b}$ (kg/m^3^) | 2160 | 2460 | 2460 | 2460 | 3000 |
| Permeability $\kappa_{11,22}$ (m^2^) horizontal | 7.5 × 10^-14^ | 1.5 × 10^-15^ | 1 × 10^-14^ | 1.5 × 10^-15^ | 5 × 10^-18^ |
| Permeability $\kappa_{33}$ (m^2^) vertical | 1.5 × 10^-14^ | 0.3 × 10^-15^ | 0.2 × 10^-14^ | 0.3 × 10^-15^ | 1 × 10^-18^ |
| Porosity $\theta$ | 0.1 | 0.1 | 0.09 | 0.07 | 0.04 |
| Fluid density $\rho$ (kg/m^3^) | 1000 | 1000 | 1000 | 1000 | 1000 |
| Fluid compressibility $\chi_{f}$ (Pa^-1^) | 4.5 × 10^-10^ | 4.5 × 10^-10^ | 4.5 × 10^-10^ | 4.5 × 10^-10^ | 4.5 × 10^-10^ |
| Rock compressibility $\chi_{p}$ (Pa^-1^) | 9.8 × 10^-11^ | 3.3 × 10^-11^ | 3.3 × 10^-11^ | 3.3 × 10^-11^ | 1.5 × 10^-11^ |
| Fluid dynamic viscosity $\mu_{d}$ (Pa·s) | 0.79 × 10^-3^ | 0.79 × 10^-3^ | 0.79 × 10^-3^ | 0.79 × 10^-3^ | 0.79 × 10^-3^ |
